# Complex renal cysts associated with crizotinib treatment

**DOI:** 10.1002/cam4.437

**Published:** 2015-03-10

**Authors:** Patrick Schnell, Cynthia H Bartlett, Benjamin J Solomon, Vanessa Tassell, Alice T Shaw, Tommaso de Pas, Soo-Hyun Lee, Geon Kook Lee, Kaoru Tanaka, Weiwei Tan, Yiyun Tang, Keith D Wilner, Allan Safferman, Ji-Youn Han

**Affiliations:** 1 Pfizer OncologyNew York, New York; 2 Peter MacCallum Cancer CentreMelbourne, Australia; 3 Pfizer OncologyLa Jolla, California; 4 Massachusetts General HospitalBoston, Massachusetts; 5 European Institute of OncologyMilan, Italy; 6 National Cancer CenterGoyang, Gyeonggi, Korea; 7 Kinki University Faculty of MedicineOsaka, Japan

**Keywords:** Crizotinib, independent radiologic review, NSCLC, renal cysts, risk factors

## Abstract

An apparent causal association between crizotinib treatment and renal cyst development emerged during clinical trials in anaplastic lymphoma kinase (*ALK*)-positive non–small cell lung cancer (NSCLC). Serious adverse event (SAE) reports of renal cysts from a safety database of 1375 patients from four clinical trials were reviewed. A blinded, retrospective, independent radiologic review (IRR) was performed using scans from patients on study for ≥6 months in three clinical trials; risk factors for renal cyst development were assessed. Among 17 patients with renal cysts reported as SAEs, evidence of invasion into adjacent structures was noted in seven patients, with no evidence of malignancy found. These patients generally did not require dose reductions, none required permanent crizotinib discontinuation due to this AE, and most continued treatment with clinical benefit. In the blinded IRR, among 255 crizotinib-treated patients, 22%, 3%, and 2% had preexisting simple cysts, complex cysts, or both, respectively. At the 6-month tumor assessment, 9% of all patients had acquired new cysts, and 2% of patients with preexisting cysts had developed new cysts and enlargements (>50%) of preexisting simple cysts. Asians appeared to have an increased risk of developing new cysts on treatment; Koreans in particular had 5.18 times higher odds of developing cysts than non-Asians (95% confidence interval, 1.51–17.78; *P *=* *0.05). Crizotinib treatment appears to be associated with an increased risk of development and progression of renal cysts in patients with *ALK*-positive NSCLC. While close monitoring is recommended, dosing modification was not generally necessary, allowing patients to remain on crizotinib treatment.

## Introduction

Crizotinib is a potent and selective small-molecule inhibitor of the receptor tyrosine kinases (RTKs) anaplastic lymphoma kinase (ALK), hepatocyte growth factor (HGF) receptor (MET), ROS1, and RON (Pfizer Inc., data on file).^1^,^2^ *ALK* gene rearrangement (*ALK*-positivity) occurs in 4% to 7% of patients with non–small cell lung cancer (NSCLC).^3^–^6^ In a randomized phase III study in previously treated *ALK*-positive NSCLC (PROFILE 1007), crizotinib treatment was shown to be superior to standard single-agent chemotherapy (pemetrexed or docetaxel) in prolonging progression-free survival.^7^ Crizotinib has now been approved in over 70 countries for the treatment of locally advanced or metastatic *ALK*-positive NSCLC.

The first serious adverse event (SAE) comprising a complex renal cyst developing during crizotinib treatment was reported in a phase II study of crizotinib 250 mg twice daily in patients with *ALK*-positive NSCLC (PROFILE 1005) in January 2011 (patient 1, Table 1). Additional cases were reported subsequently, prompting consideration of a causal association with crizotinib. Among 1205 patients who received crizotinib in the two largest crizotinib clinical trials (PROFILE 1005 and PROFILE 1007) through 10 September 2012, 24 patients (2%) had AE reports of treatment-related renal cysts. To our knowledge, this is the first time that a tyrosine kinase inhibitor has been associated with renal cyst development.

**Table 1:** Summary of cases of renal cysts reported as serious adverse events

Pt.	Demographics^1^	Baseline renal cysts	Time to diagnosis^2^ (months)	Initial symptoms	Aspiration/biopsy performed	Complication/invasion	Crizotinib dose held	Crizotinib dose reduced	Hospitalized^3^	Mean crizotinib *C*_trough,ss_ (ng/mL)
1	61F A	No	2.6	Flank pain	Aspiration Biopsy	Infiltration of R psoas muscle, anterior abdomen, and L perinephric region	Yes	No	Yes	444
2	56F A	Yes	5.5	None	Aspiration	Infiltration of para-renal space and abdominal wall	No	No	Yes	612
3^4^	59F W	Yes	6.9	None	Biopsy	−	No	No	NR	NA
4	52F A	No	3.9	Chills Fever	Aspiration	Renal abscess with L perinephric fluid collection	Yes^5^	Yes^5^	Yes	394
5	55M A	Yes	2.8	NR	Biopsy	−	No	No	NR	326
6	33F W	NR	1.2	None	Biopsy	Small bowel sub-occlusion; multiple abdominal fluid collection	Yes	No	Yes	673
7	30M A	NR	8.3	Flank pain	Biopsy	R psoas muscle	Yes	No	Yes	734
8	57M A	Yes	8.3	Fever	Aspiration	−	Yes	No	Yes	NA
9	46F A	Yes	13.8	None	NR	−	No	No	No	365
10	58M A	No	14.0	None	Biopsy	−	Yes	Yes	Yes	434
11	65F A	NR	10.9	None	Biopsy	−	Yes	No	Yes	370
12	51M A	Yes	4.1	None	NR	−	No	No	No	NA
13	53F W	Yes	3.9	None	NR	−	No	No	No	342
14	70F A	NR	5.3	None	NR	Infiltration of wall of ascending colon and L psoas muscle	Yes^6^	No	Yes	670
15	45M A	NR	15.2	None	NR	−	No	No	No	387
16	54M A	NR	6.6	None	Aspiration	Hematoma	Yes	No	Yes	590
17	65F A	Yes	10.3	None	Aspiration	Infiltration of duodenal wall and R perinephric space	Yes	Yes	Yes	NA

A, Asian; *C*_trough,ss_, steady-state trough concentration; F, female; L, left; M, male; NA, not available; NR, not reported; pt, patient; R, right; W, white.

1 Numbers denote ages of patients in years.

2 Per serious adverse event report.

3 In some cases several times.

4 Two serious adverse event reports were submitted for this patient.

5 Due to alanine aminotransferase elevation as well.

6 Due to gastrointestinal bleed as well.

Cysts are the most common space-occupying lesion of the kidneys and are classified as either simple or complex. Simple cysts are round or oval in shape and are typically lacking internal echoes, calcification, or enhancement.^8^ Septations, if present, are limited to one or two.^9^ Simple cysts are usually asymptomatic, are frequently diagnosed incidentally, and are commonly encountered in the general population, with reported prevalences of up to 41%.^10^ The prevalence, size, and bilaterality of renal cysts increases with advancing age, and cysts tend to be more common in men than women.^10^,^11^ At least some of the differences in the prevalence rates reported over time may be due to the recent emergence of more advanced imaging techniques.^12^

Complex renal cysts are relatively uncommon (prevalence <1%)^13^,^14^ and often irregular in shape, typically have multiple septae, and may show evidence of inflammatory stranding, calcification, and contrast enhancement.^15^ The Bosniak classification system distinguishes five categories of renal cysts based on their appearance and enhancement on computed tomography (CT; categories I and II correspond to simple cysts; categories IIF, III, and IV correspond to complex cysts with increasing potential for malignancy^16^). The incidence rate of complex renal cysts in patients with NSCLC is unknown.

## Methods

The first reported case of renal cysts is presented. SAE reports from a safety database of four ongoing company-sponsored clinical trials (PROFILE 1001, a phase I study;^17^,^18^ PROFILE 1005;^19^ PROFILE 1007;^7^ and PROFILE 1014, a randomized phase III study^20^) consistent with renal cysts developing in crizotinib-treated patients (starting dose, 250 mg twice daily) with *ALK*-positive NSCLC were evaluated through 31 December 2012. Pertinent information was extracted, and an aggregate analysis was performed. Available tumor response data for patients with these SAEs, as recorded in the clinical database and assessed through 31 December 2012, were also evaluated.

To determine the incidence of renal cyst development or enlargement of preexisting renal cysts during crizotinib treatment independent of AE reporting, an independent radiology laboratory (Core Lab Partners, Princeton, NJ) performed a blinded retrospective review of CT and magnetic resonance imaging (MRI) scans from patients on study for at least 6 months as of August 2011. Available scans were obtained from three studies: PROFILE 1001, PROFILE 1005, and PROFILE 1007 (scans were not included from PROFILE 1014 because this study was still blinded and enrolling patients at the time this analysis was undertaken). Imaging (CT or MRI) in these three studies was carried out at screening, every 6 weeks (PROFILE 1005 and 1007) or every 8 weeks (PROFILE 1001), and whenever disease progression was suspected, as specified in the respective protocols. Use of contrast agents was required unless medically contraindicated, in which case MRI examinations were performed. If renal cysts were detected by the independent radiologic review (IRR) at the time of screening or at the protocol-specified 6-month tumor assessment after start of treatment or randomization, all scans were evaluated to assess the time of first appearance and to monitor the evolution of the cyst(s) over time using the updated Bosniak classification system.^16^

Blood samples were collected on day 1 of treatment cycles 1 and 2 (and day 15 of cycles 1 and 2 for some patients) and on day 1 of cycles 3 and/or 5 in the three studies. Plasma crizotinib concentrations were determined using validated methods involving high-performance liquid chromatography with tandem mass spectrometry. The arithmetic mean steady–state crizotinib trough concentration (*C*_trough,ss_) in each patient was calculated using all evaluable predose plasma concentrations (*C*_trough_) from cycle 1 day 15 through cycle 5 day 1, wherever available.

Logistic regression models were used to investigate the potential association between the risk of developing on-study renal cysts and factors comprising age, gender, ethnicity, *C*_trough,ss_ (log-transformed), and creatinine clearance. Because of the small sample size, backward model selection techniques were employed. Data cutoffs for these analyses were 13 April 2012 for PROFILE 1001, 15 February 2012 for PROFILE 1005, and 30 March 2012 for PROFILE 1007.

## Results

### Case presentation

**Figure 1:**
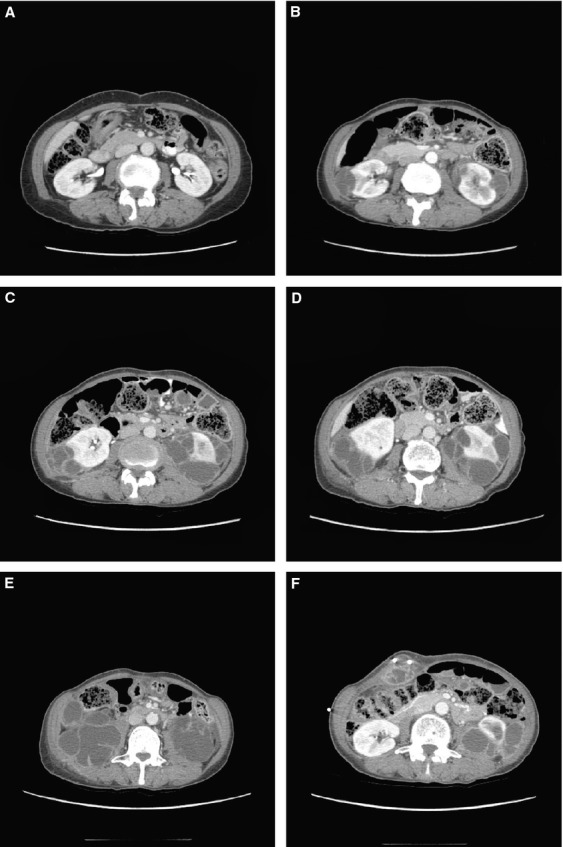
Computed tomography images of a 61-year-old woman with *ALK*-positive NSCLC who developed complex renal cysts during treatment with crizotinib (patient 1, Table 1). (A) baseline and (B) 2.5 months, (C) 4.1 months, (D) 5.5 months, (E) 6.3 months (just prior to cyst resection), and (F) 18.1 months after initiation of treatment. The images show progressive growth of multiseptated renal cystic lesions infiltrating the perirenal space and involving the left psoas muscle. Additionally, a cystic lesion in the anterior abdominal wall developed (drain in place, F), which was resected. Pathology showed degenerated muscular tissue with fibrosis and acute inflammation. NSCLC, non–small cell lung cancer. ALK, anaplastic lymphoma kinase.

**Figure 2:**
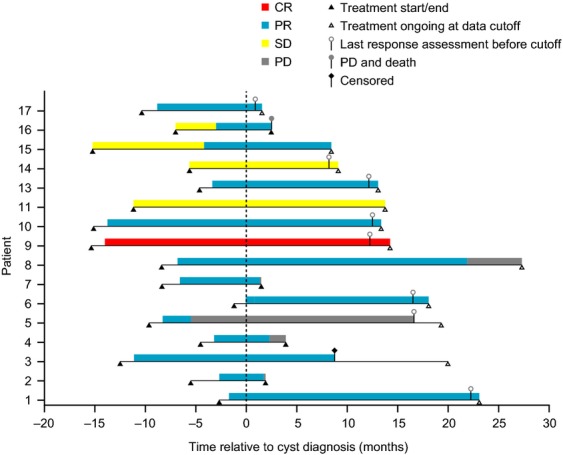
Treatment and responses at data cutoff (31 December 2012) in patients with renal cysts reported as serious adverse events. Cyst diagnosis dates were based on radiologic assessment at the study sites. Derived response data are depicted per RECIST. Relevant data were not available for patient 12. RECIST, response evaluation criteria in solid tumors.

The first SAE report of a complex renal cyst in the crizotinib development program involved a 61-year-old (42-kg) Asian woman with *ALK*-positive NSCLC (patient 1, Table 1). The patient, who had no underlying renal disease, normal baseline renal imaging scans (Fig. 1A), and no history of trauma, developed left-sided flank pain 2.6 months after starting crizotinib treatment. The patient was hospitalized, and a pelvic CT scan revealed well-enhanced multiseptated cystic lesions in both kidneys encroaching into the perirenal space (Fig. 1B). Blood creatinine, urinalysis, and urine culture were normal. Crizotinib was held for several days and reinitiated upon symptomatic improvement. Follow-up CT scans performed ∽2 and 3 months later (Fig. 1C and D) revealed an increase in renal lesion size (≤5 cm), with a more significant presence in the perirenal space. Cyst aspiration was negative for malignant cells. Approximately 4 months after restarting crizotinib, the cystic masses extended into the right psoas and quadratus lumborum. An open biopsy performed in the left perirenal area was consistent with xantho-granulomatous perinephritis without evidence of malignancy and with negative cultures. A percutaneous drain was placed; however, the patient presented at the hospital with abdominal pain and general weakness soon thereafter. Examination revealed a nontender elongated movable mass, and an abscess of the right anterior abdominal wall, as well as an increase in the right inferior cystic renal lesion to 6 cm was diagnosed; the previously diagnosed lesions in the left psoas and back muscles were stable. Over the following 18 months, the patient underwent multiple surgical interventions and drainage procedures related to locally invasive cystic masses (Fig. 1E and F), although there was limited systemic involvement, no evidence of malignancy, and no renal impairment. Crizotinib treatment was ongoing at data cutoff, 25.8 months after initiation. The patient had a partial response (PR) as defined using response evaluation criteria in solid tumors (RECIST) that began 1.2 months prior to cyst diagnosis and had persisted through data cutoff, 23.3 months after cyst diagnosis (Fig. 2).

### Signs, symptoms, and laboratory findings (SAE reports)

Of 17 patients with renal cysts reported as SAEs (Table 1), median age was 55 years, 59% were female, and 82% were Asian, of whom 43% were Korean. In comparison, among 1375 patients treated with crizotinib (any dose) for *ALK*-positive NSCLC in clinical trials, median age was 52 years, 56% were female, and 44% were Asian, of whom 30% were Korean. Among the 17 patients with renal cysts reported as SAEs, none had a reported history of polycystic kidney disease or other renal disease, although eight had preexisting simple cysts, and one patient (patient 12, Table 1) had a complex cyst at baseline. The median time to cyst diagnosis was 6.6 months (range, 1.2–15.2 months) after initiation of crizotinib treatment. Two patients presented with flank pain, and two had chills and/or fever, although it was not always clear whether the fever was incidental or related to renal cysts. Most patients (12 of 17), however, were asymptomatic. Inflammatory parameters (white blood cell counts, C-reactive protein, and sedimentation rate) were elevated in some cases. Urinalysis was normal in 10 patients and either not done or not reported in five patients. In 1 case, urine protein was marginally elevated, and in another, several abnormalities were seen; however, all resolved at the next follow-up visit. Creatinine and blood urea nitrogen (BUN) were transiently elevated in one patient with a concurrent gastrointestinal bleed, and creatinine only was marginally elevated in a second case in the context of a febrile illness. These abnormalities resolved on follow-up. Both creatinine and BUN were normal in the remainder of the patients, and none developed new or worsening of hypertension (preexisting in two cases) or renal impairment. Twelve patients underwent aspiration and/or biopsy, with no evidence of primary renal malignancy, NSCLC metastasis, or identifiable microorganisms.

### Clinical course and follow-up

Overall, 11 of 17 patients with renal cysts were known to be hospitalized due to this SAE. Evidence of cystic invasion into adjacent structures in the form of inflammatory cystic masses was noted in seven patients; percutaneous drainage of collected fluid was required in four patients. Crizotinib treatment was temporarily discontinued in 10 patients for up to 42 days; in some patients, dosing was interrupted repeatedly due to drainage procedures. Dose reductions were implemented in three patients (in 1 case due to a concurrent increase in liver enzymes). In all others, treatment was continued without modification. In 1 case (patient 2, Table 1), crizotinib was discontinued soon after diagnosis of a complex renal cyst, since renal imaging findings were consistent with possible NSCLC metastasis (presence of new cystic nodule); however, this was not confirmed histologically. Most were able to continue crizotinib treatment following a renal cyst diagnosis (Fig. 2). Of the 16 patients with available response data, 1 achieved a complete response, 13 achieved a PR, and 2 maintained stable disease as best responses (Fig. 2).

### Independent radiologic review

A blinded retrospective review of available imaging studies was performed in 272 patients enrolled in PROFILE 1001 (*n* = 85), PROFILE 1005 (*n* = 134), or PROFILE 1007 (*n* = 53, of whom 17 patients had been treated with chemotherapy [pemetrexed or docetaxel]). All of these patients had been on study for at least 6 months and had imaging scans providing at least 90% visualization of both kidneys. Among the 255 crizotinib-treated patients, 55 (22%) had preexisting simple cysts, 8 (3%) had preexisting complex cysts, and 4 (2%) had both simple and complex cysts at baseline, for an overall prevalence of 26% (Table 2). At the time of the 6-month tumor assessment, 24 patients (9%) had acquired new cysts of any type. In addition, 5 patients with preexisting cysts (2%) had developed new cysts as well as an increase in the longest diameter of preexisting cysts by >50% from baseline. Categorization of renal lesions using the Bosniak system showed that lesions of variable complexity were observed both at baseline and during crizotinib treatment (Supplemental Table 1).

**Table 2:** Frequencies of simple and complex renal cysts at baseline and on treatment in three clinical trials included in the IRR^1^

	Crizotinib (*n* = 255)				Chemotherapy (*n* = 17^2^)			
		On treatment				On treatment		
	BL	>50% size increase vs. BL only	Newly acquired only	Both >50% size increase vs. BL and newly acquired	BL	>50% size increase vs. BL only	Newly acquired only	Both >50% size increase vs. BL and newly acquired
BL cyst type	*N* (%)	*N* (%)	*N* (%)	*N* (%)	*N* (%)	*N* (%)	*N* (%)	*N* (%)
Simple cysts	55 (22)	0 (0)	8 (3)	4 (2)	4 (24)	0 (0)	0 (0)	0 (0)
Complex cysts	8 (3)	0 (0)	0 (0)	1 (<1)	1 (6)	0 (0)	0 (0)	0 (0)
Both simple and complex cysts	4 (2)	0 (0)	1 (<1)	0 (0)	0 (0)	0 (0)	0 (0)	0 (0)
None	188 (74)	NA (NA)	15 (6)	NA (NA)	12 (71)	NA (NA)	NA (NA)	NA (NA)
Total	255 (100)	0 (0)	24 (9)	5 (2)	17 (100)	0 (0)	0 (0)	0 (0)

BL, baseline; IRR, independent radiologic review; NA, not applicable.

1 PROFILE 1001, PROFILE 1005, and PROFILE 1007.

2 Only 17 chemotherapy-treated subjects met the criteria for evaluation (availability of imaging scans providing ≥90% visualization of both kidneys at screening and at the 6-month tumor assessment) since many developed progressive disease prior to completing 6 months on study.

### Evaluation of risk factors

Crizotinib plasma *C*_trough,ss_ was available for 13 of 17 patients with renal cysts reported as SAEs (Table 1). The crizotinib *C*_trough,ss_ for these 13 patients (median, 434 ng/mL; range, 326–734 ng/mL) appeared to be above the median *C*_trough,ss_ obtained in a population of 801 crizotinib-treated patients with *ALK*-positive NSCLC from PROFILE 1005 and PROFILE 1007 for whom crizotinib plasma concentrations were available (307 ng/mL). Crizotinib *C*_trough,ss_ was therefore further evaluated as a potential risk factor for the development of new renal cysts along with several other factors in 193 of the 255 patients included in the blinded IRR of available imaging studies who had available *C*_trough,ss_ values (Table 3). In this subset of patients, Asians appeared to have an increased risk of developing renal cysts on treatment, among whom Koreans had 5.18 times higher odds (95% confidence interval [CI], 1.51–17.78; *P *=* *0.05) than non-Asians (Table 4). Non-Korean Asians had 3.10 times higher odds (95% CI, 0.64–14.98; *P *=* *0.66) than non-Asians, although this observation did not reach statistical significance, and non-Koreans comprised a small subset of Asian patients in this study (Table 3). Based on the results in Table 4, the odds of developing renal cysts increased by a factor of 1.3 (95% CI, 0.99–1.66) for every 5-year increment in age or 1.05 (95% CI, 0.997–1.11) for every 1-year increment (*P *=* *0.06). The odds of developing renal cysts increased by a factor of 5.73 for every natural log-ng/mL increment in *C*_trough,ss_ (95% CI, 0.90–36.39; *P *=* *0.06). For example, a patient with a *C*_trough,ss_ of 380 ng/mL (median *C*_trough,ss_ for patients who developed new renal cysts) would be predicted to have 1.5 times higher odds (95% CI, 0.98–2.28; *P *=* *0.06) of developing renal cysts than a patient with a *C*_trough,ss_ of 302 ng/mL (median *C*_trough,ss_ for patients who did not develop new cysts). However, in logistic regression analysis within each subgroup defined by ethnicity, neither age nor *C*_trough,ss_ were significant factors. Similar results were obtained using forward or stepwise model-selection processes.

**Table 3:** Baseline characteristics and plasma crizotinib concentrations of patients^1^ by renal cyst status at baseline and on crizotinib treatment

	Development of renal cysts on crizotinib		No development of renal cysts on crizotinib	
	Newly acquired on treatment only (*n* = 11)	Preexisting and newly acquired on treatment (*n* = 7)	No cysts at any time (*n* = 135)	Preexisting at baseline only (*n* = 40)
Characteristic	*N* (%)	*N* (%)	*N* (%)	*N* (%)
Age (years), median (range)	48 (41–67)	61 (55–78)	50 (22–79)	58 (30–79)
Age ≥65 years	1 (9)	3 (43)	11 (8)	10 (25)
Male	4 (36)	5 (71)	52 (39)	21 (52)
Ethnicity				
White	3 (27)	2 (29)	78 (58)	28 (70)
Asian	8 (73)	5 (71)	49 (36)	9 (22)
Japanese	0 (0)	1 (14)	9 (7)	2 (5)
Korean	7 (64)	3 (43)	26 (19)	7 (18)
Chinese	0 (0)	1 (14)	11 (8)	0 (0)
Other Asians	1 (9)	0 (0)	3 (2)	0 (0)
Black	0 (0)	0 (0)	4 (3)	0 (0)
Other	0 (0)	0 (0)	4 (3)	3 (8)
Smoking history				
Never-smoker	8 (73)	3 (43)	102 (76)	27 (68)
Former smoker	3 (27)	3 (43)	31 (23)	12 (30)
Current smoker	0 (0)	1 (14)	2 (1)	1 (2)
Weight (kg), median (range)	61 (46–117)	65 (50–80)	66 (38–151)	69 (36–103)
Renal impairment				
Normal	5 (45)	2 (29)	72 (53)	19 (48)
Mild	5 (45)	2 (29)	49 (36)	18 (45)
Moderate	1 (9)	3 (43)	8 (6)	3 (8)
Not reported	0 (0)	0 (0)	6 (4)	0 (0)
*C*_trough_ (ng/mL), geometric mean (% CV)	389 (27)	370 (23)	290 (37)	316 (36)

*C*_trough_, trough plasma concentration; CV, coefficient of variation.

1 Patients in the blinded independent radiologic review with available crizotinib *C*_trough_ values.

**Table 4:** Potential association between demographic characteristics and crizotinib exposure, and risk of developing renal cysts on crizotinib treatment: logistic regression analysis^1^

	OR^2^	95% CI	*P* 3
Age (1-year increments)^4^	1.05	0.997–1.11	0.06
Race (Korean vs. non-Asian)	5.18	1.51–17.78	0.05
Race (non-Korean Asian vs. non-Asian)	3.10	0.64–14.98	0.66
Ln mean *C*_trough,ss_ (ln ng/mL increments)^4^	5.73	0.90–36.39	0.06

*C*_trough,ss_, arithmetic mean steady-state crizotinib trough concentration; ln, natural log; OR, odds ratio.

1 Final variables for the model were selected using a backward selection process and a two-sided alpha level of 0.10. Factors used in model selection were age (years), sex (male vs. female), race (Korean vs. non-Asian, non-Korean Asian *vs*. non-Asian), creatinine clearance (mL/min), and ln mean crizotinib *C*_trough,ss_ (ln ng/mL).

2 For categorical variables, OR > 1 indicates higher odds of developing renal cysts in the first category; for continuous variables, OR > 1 indicates that the OR increases as the variable increases.

3 Wald chi-square test.

4 Continuous variable.

## Discussion

The incidence or prevalence of simple or complex renal cysts in patients with either lung cancer or *ALK*-positive NSCLC has not previously been systematically evaluated. A recent case report described the development of a complex renal cyst about 6 months after the start of crizotinib treatment in a 49-year-old female with *ALK*-positive NSCLC.^21^ Crizotinib was continued due to ongoing clinical benefit, and the complex renal cyst regressed 22 months later despite continuing treatment. In a retrospective analysis of 32 Taiwanese patients with *ALK*-positive NSCLC treated with crizotinib and followed up for a median of 493 days, significant cystic changes in the kidneys were observed in 7 patients (22%), most of which regressed after discontinuation of crizotinib.^22^

The results of the IRR conducted in 272 patients (255 treated with crizotinib and 17 with chemotherapy) from three clinical trials presented here represent the first large-scale systematic examination of the incidence of renal cysts in patients with *ALK*-positive NSCLC treated with crizotinib. Patients with *ALK*-positive NSCLC tend to be relatively young (median age ∽50 years).^23^ The prevalence of simple cysts observed in this population (22%) is comparable to the rate in the general population in this age group.^10^ However, 5% of patients had preexisting complex renal cysts, suggesting that there may be a considerably higher prevalence of complex renal cysts in patients with *ALK*-positive NSCLC than in the general population. Nevertheless, given that among 255 patients evaluated at the 6-month tumor assessment 9% had developed new cysts, and 2% had both new cysts and size increases in existing cysts of more than 50%, a causal role of crizotinib in cyst development is plausible, particularly since none of the 17 patients randomized to chemotherapy treatment with available scans developed new cysts. Crizotinib not only appeared to be associated with new renal cyst formation, it may also have played a role in facilitating the progression of existing small or subclinical cystic lesions.

Crizotinib plasma *C*_trough,ss_ was not found to be a significant driver of new renal cyst development based on a logistic regression model involving 193 of the 255 patients with blinded IRR imaging after adjusting for age and ethnicity, indicating that renal cyst development may not be associated with higher plasma exposure of crizotinib. Although 13 of 17 patients with renal cysts reported as SAEs appeared to have a higher median crizotinib *C*_trough,ss_ than that of the general population of patients with *ALK*-positive NSCLC, 11 were Asian patients, whose plasma crizotinib exposure is known to be generally higher than that of non-Asians.^24^ Of note, six of 17 patients with renal cysts reported as SAEs were Korean (35%), and Korean ethnicity was the only factor that was found to be significantly associated with development of new renal cysts on crizotinib treatment in the IRR patient population in the logistic regression model (of 193 patients included in this model, 71 were Asians, of whom 43 were Korean). Due to the small sample size, however, these results should be interpreted with caution; this analysis could not distinguish whether the increased risk of developing renal cysts in Koreans was due only to ethnicity or whether other factors, such as age, gender, renal function, or crizotinib exposure were also involved. However, in the time since the IRR was performed, results of PROFILE 1014 have become available.^20^ AE data from this study support these observations (Pfizer Inc., data on file): among 171 patients treated with crizotinib (for a median duration of 47.4 weeks), renal cysts were reported in six of 77 Asians (8%): four of 30 Koreans (13%), one of 27 Chinese (4%), and 0 of 14 Japanese. One renal cyst was reported among six Asians whose geographic origin was not further specified. Among 94 crizotinib-treated non-Asians, renal cysts were reported in two patients (2%). Furthermore, in PROFILE 1014, an SAE of abdominal abscess originating from bilateral complex renal cysts was reported in a crizotinib-treated 69-year-old white female about 1 year after start of treatment with crizotinib. Among 169 patients treated with chemotherapy (median duration, 18.0 weeks), a renal cyst was reported in 1 Chinese patient (1%). Additionally, three Asian patients (1 Korean, 1 Japanese, and 1 Chinese) developed renal cysts after crossing over from chemotherapy to receive crizotinib treatment within the study.

The mechanisms by which crizotinib may increase the risk of developing renal cysts are currently unknown. Crizotinib is a potent ALK, MET, ROS1, and RON inhibitor (Pfizer Inc., data on file).^1^,^2^ HGF, the only known ligand for MET, is involved in embryonic development, tissue regeneration, and tumor progression.^25^ In the kidney, HGF is expressed in mesangial and interstitial stromal cells, which are mesenchymal, while MET is expressed in nonmesenchymal cells, such as those of the tubular epithelium. A limited number of reports have implicated activation of the HGF−MET signaling axis in the genesis of renal cysts,^26^,^27^ and this pathway has been identified as a possible therapeutic target in renal cell carcinoma.^25^,^28^ However, these observations would suggest that crizotinib should inhibit, rather than induce, cyst formation and therefore do not provide a mechanism of renal cyst formation associated with crizotinib treatment.

The degree of complexity of renal cysts has been shown to correlate with their malignant potential.^11^,^16^ Although in our analyses work-up for renal malignancy was limited to aspiration, and biopsies were not performed in all cases, no evidence of malignancy was found based on diagnostic testing (including follow-up with imaging scans) or clinical follow-up in any of the crizotinib-treated patients diagnosed with complex renal cysts (Bosniak categories III and IV). Patients who developed renal cysts generally did not require dose reductions, and no patients required permanent discontinuation of crizotinib treatment due to this AE. Continued treatment of *ALK*-positive NSCLC with crizotinib despite development of renal cysts was associated with ongoing tumor control, since RECIST-defined responses were maintained for a median of 14.0 months from treatment initiation in the 14 patients with objective responses.

In almost half of the SAE cases presented, complex renal cysts expanded into adjacent extrarenal spaces and, in most of those cases, percutaneous drainage became necessary. Close monitoring of patients who have preexisting renal cysts or who develop cysts on crizotinib treatment is therefore recommended. This should include appropriate imaging at regular intervals and may include expert consultation if necessary. However, given that no primary or secondary malignancies have been identified in these or in the IRR population to date, standard guidelines recommending surgery for category III and IV lesions^29^ may not apply in this group of patients.

In summary, while it is possible that *ALK*-positive NSCLC in itself may constitute a major risk factor for the development of complex renal cysts, treatment with crizotinib appears to be associated with an increased risk for the development and progression of renal cysts in patients with *ALK*-positive NSCLC. In some cases, this led to cystic invasion into adjacent tissue requiring drainage. While close monitoring is recommended, dosing modification was not generally necessary, allowing patients to continue to benefit from crizotinib treatment.

## Supporting Information

**Table S1.** Bosniak classification of renal cysts in crizotinib-treated patients.
