# Supplementary material for: Complex renal cysts associated with crizotinib treatment
Source: Cancer Med. 2015 Mar 10;4(6):887–96. doi: 10.1002/cam4.437 (PMC4472211; doi:10.1002/cam4.437)
Supplement: Supplementary file 1 [file cam40004-0887-sd1.docx]

SUPPLEMENTARY DATA

| **Supplemental Table 1.** Bosniak Classification of Renal Cysts in Crizotinib-treated Patients^a^ | | | |
| --- | --- | --- | --- |
| Bosniak Classification | Patients With Renal Cysts at BL | | No. of Patients Without BL Renal Cysts Who Developed  New Cysts Only^b^ |
|  | No. of Patients | No. of Patients Who Developed New Cysts and/or Higher Bosniak Classifications^b^ |  |
| I | 40 | 6 | 7 |
| II | 16 | 2 | 1 |
| IIF | 2 | 1 | 4 |
| III | 5 | 6 | 3 |
| IV | 3 | 0 | 0 |
| Unevaluable | 1 | 0 | 0 |
| Total | 67 | 15 | 15 |
| BL, baseline.  ^a^ Among 255 crizotinib-treated patients in the independent radiologic review.  ^b^ At the time of the 6-month tumor assessment. | | | |
